# Efficacy of Virtual Reality–Based Interventions on Cognitive Function in Patients With Neuropsychiatric Disorders: Systematic Review and Meta-Analysis of Randomized Controlled Trials

**DOI:** 10.2196/67501

**Published:** 2025-05-08

**Authors:** Qiujing Du, Yuhan Wei, Yuexuan Ma, Changqing Liu, Shanshan Du, Qi Zhang, Xiaotong Gong, Jiaju Yang, Qijie Li, Ka Li

**Affiliations:** 1 Medicine and Engineering Interdisciplinary Research Laboratory of Nursing & Materials, West China Hospital/West China School of Nursing, Sichuan University, 37 Guoxue Lane, Wuhou District, Chengdu, China, 86 18980601488, 86 85421125; 2 West China Hospital/West China School of Nursing, Sichuan University, Chengdu, China

**Keywords:** virtual reality, neuropsychiatric disorders, cognitive function, systematic review, meta-analysis, mobile phone

## Abstract

**Background:**

Virtual reality (VR) technology has emerged as a promising tool for cognitive rehabilitation in patients with neuropsychiatric disorders. These patients often endure significant cognitive impairments, which are associated with decreased quality of life and increased disease burden. Traditional treatments are limited in improving cognitive functions, making VR-based interventions an area of growing interest.

**Objective:**

This meta-analysis aims to evaluate the efficacy of VR-based interventions on cognitive function in patients with neuropsychiatric disorders by synthesizing data from randomized controlled trials (RCTs).

**Methods:**

Following PRISMA guidelines, we conducted a comprehensive search across PubMed, Web of Science, MEDLINE, EMBASE, and Cochrane Library for RCTs from January 2010 to December 2024. Studies were included if they evaluated the impact of VR-based interventions on cognitive outcomes in patients with neuropsychiatric disorders. Data extraction and risk of bias assessment were performed independently by 2 researchers. Meta-analyses were conducted using random-effects models, and standardized mean differences (SMDs) as effect size.

**Results:**

A total of 21 RCTs involving 1051 participants were included. Overall, VR-based interventions significantly improved cognitive functions of patients with neuropsychiatric disorders (SMD 0.67, 95% CI 0.33-1.01, *z*=3.85; *P*<.001). Subgroup analyses revealed significant benefits for cognitive rehabilitation training (SMD 0.75, 95% CI 0.33-1.17, *z*=3.53; *P*<.001), exergame-based training (SMD 1.09, 95% CI 0.26-1.91, *z*=2.57; *P*=.01), and telerehabilitation and social functioning training (SMD 2.21, 95% CI 1.11-3.32, *z*=3.92; *P*<.001). Conversely, immersive cognitive training, music attention training, and vocational and problem-solving skills training did not yield significant improvements (*z*=1.86, *P*=.06; *z*=0.35, *P*=.72; *z*=0.88, *P*=.38; respectively). Disease-type subgroup analyses indicated significant improvements in schizophrenia (SMD 0.92, 95% CI 0.22-1.62, *z*=2.58; *P*=.01), and mild cognitive impairment (SMD 0.75, 95% CI 0.16-1.35, *z*=2.47; *P*=.01), but not in brain injuries, Parkinson disease, or stroke (*z*=0.34, *P*=.73; *z*=1.26, *P*=.21; *z*=1.16, *P*=.24; respectively).

**Conclusions:**

This meta-analysis revealed that VR-based interventions can improve cognitive functions among individuals with neuropsychiatric disorders, with notable improvements observed in cognitive rehabilitation training, exergame-based training, and tele-rehabilitation and social functioning training. These results offer valuable evidence supporting the use of VR technology in rehabilitation for neuropsychiatric conditions and inform the optimization of future intervention approaches.

## Introduction

“Patients with neuropsychiatric disorders” refer to individuals enduring neurological or psychiatric conditions, or those exhibiting symptoms pertinent to both neurology and psychiatry [1]. The illnesses of these patients typically involve dysfunction within the nervous system (including the brain), accompanied by psychological or behavioral abnormalities [2]. Cognitive impairment, a prevalent symptom in these patients with an incidence exceeding 70%, is characterized by a marked and measurable decline in cognitive abilities compared to previous levels of function. This can manifest as deficits in higher-order brain functions such as learning, memory, problem-solving, and decision-making [3,4]. Research has confirmed that the occurrence of cognitive impairments in patients with neuropsychiatric disorders is significantly associated with decreased quality of life and increased disease burden [5,6], which may lead to more complex health challenges and heightened care requirements, imposing substantial pressure on the patients themselves, their families, and society at large. Therefore, there is an urgent need to develop relevant strategies to address these significant challenges, aiming to improve cognitive function and enhance the quality of life for these patients.

Traditional intervention strategies for cognitive function in patients with neuropsychiatric disorders primarily include pharmacological treatment and cognitive rehabilitation. Pharmacological treatments are commonly used to alleviate psychiatric symptoms but have limited efficacy in improving cognitive functions [7]. Cognitive rehabilitation is considered a promising nonpharmacological intervention, aiming to enhance patients’ cognitive functions through systematic training and practice. Studies have shown that cognitive rehabilitation can effectively improve cognitive domains such as attention, executive function, and working memory in these patients [8]. However, the implementation of traditional cognitive rehabilitation may face challenges in resource-limited settings, and the transfer effect of cognitive enhancements to functional results in individuals’ everyday lives is generally insufficient. Moreover, a lack of motivation among participants represents one of the key barriers to achieving effective therapeutic goals in traditional cognitive rehabilitation [9,10].

Virtual reality (VR) and augmented reality (AR) are emerging technologies with significant potential to address the challenges associated with cognitive rehabilitation. VR technology is an advanced system that integrates information and computer technologies to create lifelike web-based environments using computer-generated imagery and digital interactive devices. It is designed to provide users with an immersive experience, enabling them to not only observe passively but to also interact actively with the digital environment [11]. VR technology could provide a fully controllable and secure setting, supporting remote access and offering a more ecologically valid and effective environment for cognitive rehabilitation [12]. Second, it can simulate multimodal scenarios highly similar to those that patients might encounter in their daily lives, making it easier to integrate cognitive training with everyday functioning [13]. Third, its high level of interactivity and game-inspired design can help increase patient participation [14]. Compared to VR, fewer studies have explored the use of AR in cognitive rehabilitation. AR, by integrating virtual elements with the physical world, carries a lower risk of adverse events, such as falls or dizziness, making it a safer alternative in some contexts [15]. However, immersive VR provides a higher level of engagement and cognitive stimulation, which is particularly beneficial in exergame-based training. To address the safety concerns associated with immersive VR, specific measures are implemented, such as having medical personnel present during sessions and limiting session duration to mitigate dizziness [16-18]. These precautions ensure the safe and effective use of immersive VR in cognitive rehabilitation. A recent meta-analysis has shown that VR-based interventions have a beneficial effect on overall cognition and executive function in older adults [19]. Additionally, a previous narrative review indicated promising results of VR-based cognitive rehabilitation in executive function and visuospatial abilities [20], nevertheless, this review focused on patients with neurological disorders and did not conduct a quality assessment or quantitative synthesis of the studies reviewed. Moreover, different types of cognitive rehabilitation interventions have varying impacts on cognitive functions, and their interaction effects with VR technology differ [21].

Based on this, our study consolidates data from randomized controlled trials (RCTs) of VR-based interventions aimed at improving cognitive function in patients with neuropsychiatric disorders, to evaluate the efficacy of interventions and quality of these RCTs. The objectives of our study are (1) to quantitatively investigate the efficacy of VR-based interventions on the cognitive function of patients with neuropsychiatric disorders and (2) to assess the effects of different types of interventions and analyze possible reasons for any differences observed.

## Methods

### Study Design

The design, execution, and reporting of this meta-analysis adhered to the PRISMA guidelines of the 2020 statement [22]. This study has been registered at PROSPERO, and the registration number is CRD42023445000.

### Ethical Considerations

No humans or animals were involved in this meta-analysis, so ethical approval was waived.

### Search Strategy

An exhaustive search was systematically performed across multiple databases, including PubMed, Web of Science, MEDLINE, EMBASE, and Cochrane Library. In light of the progressive maturation of VR technology subsequent to 2010 and the increased scholarly attention toward exploring VR as a modality for supporting psychological and cognitive interventions during this period [23], the timeframe for our literature search shall be delimited from January 2010 to December 2024. The used search methodology was grounded in the PICOS framework, encompassing the specific elements of Patient or Population, Intervention, Comparison, Outcome, and Study type. No specific restrictions were applied regarding demographic variables such as age, gender, education, etc. However, for the language, the search was conducted exclusively in the English language. The search methodology used multiple strategies, including examination of titles and abstracts, use of relevant keywords, and incorporation of free and medical subject headings terms, etc. The specific retrieval strategies for each database are outlined in Multimedia Appendix 1.

### Criteria for Study Eligibility

Inclusion criteria for our review encompassed the following criteria: (1) participants were diagnosed with neuropsychiatric diseases; (2) interventions used the VR technology; (3) the control groups were assigned to either receive conventional treatment or were placed on a waitlist for comparison purposes; (4) the study quantitatively assessed the cognitive function. No restrictions were imposed on the specific measurement tools or instruments used to evaluate the indices; (5) the literature was published in English; and (6) the included studies were designed per the principles of RCTs. Exclusion criteria for this study encompassed the following criteria: (1) qualitative study, conference paper, letter, review, commentary, or protocol; or (2) studies where access to full-text articles was not available or where relevant full data were not available.

### Study Selection and Data Extraction

All identified study titles and abstracts were retrieved and imported into Zotero 7.0 (Corporation for Digital Scholarship). Duplicate papers were subsequently removed from the dataset. Two researchers independently assessed the titles and abstracts of the studies in parallel to ensure compliance with the inclusion criteria. Subsequently, the full texts of each study were reviewed per the predetermined eligibility criteria. Discrepancies were resolved through a consensus-seeking process involving discussion and consultation with a third investigator.

Two researchers independently performed data extraction from the selected studies and documented the relevant information within a pre-established data extraction form, encompassing the following essential components: the first author, the published year, the country or region where the trial was conducted, the disease type, the ages (mean and SD), the years of education (mean and SD); the sample size (experimental or control group), and the gender; the type, content, hardware, software, immersive or nonimmersive approach and detailed session information of the intervention; and the assessment time and the instruments of outcome measures.

### Quality Assessment

Using the Cochrane RoB2 (Risk of Bias 2) tool, 2 investigators carried out a separate evaluation of the methodological rigor and potential bias of the selected studies. RoB2 is a systematic review tool developed by the Cochrane Risk of Bias Working Group aimed at assessing the methodological quality of RCTs [24]. This tool identifies potential sources of bias that may affect the reliability of study outcomes through a series of guiding questions that cover key areas such as the randomization process, deviations from intended interventions, missing outcome data, outcome measurement, and selective reporting. It provides a final judgment on the risk of bias. Third-party consultation was used to resolve disagreements.

### Data Synthesis and Analysis

We used Review Manager 5.3 (Cochrane) to conduct our statistical analyses. For continuous outcome indexes, the mean difference along with a 95% CI was used when all selected studies reported outcomes using the same unit and magnitude; otherwise, the standard mean difference was used to express the results.

Initially, the chi-square test was used to assess the statistical heterogeneity among the outcomes reported in each individual study, accompanied by the calculation of the corresponding *P* value; to quantitatively evaluate the magnitude of heterogeneity, the *I*^2^ test was used [25]. Given the likelihood of functional disparities among studies conducted independently by different researchers, a random-effects meta-analysis was conducted for the outcome to account for potential heterogeneity across the included studies. Additionally, we conducted a subgroup analysis based on different types of interventions. To explore the potential reasons for the differences in the integrated outcomes of various intervention types based on VR technology, we conducted another subgroup analysis for different types of neuropsychiatric disorders and carried out sensitivity analyses by excluding certain studies. We constructed a funnel plot to evaluate potential publication bias systematically across the studies. In the final stage, forest plots were generated to visually present the effect estimates of outcome variables across all included studies. A level of statistical significance was established at *P*<.05.

## Results

### Search Results and Selection

**Figure 1.**
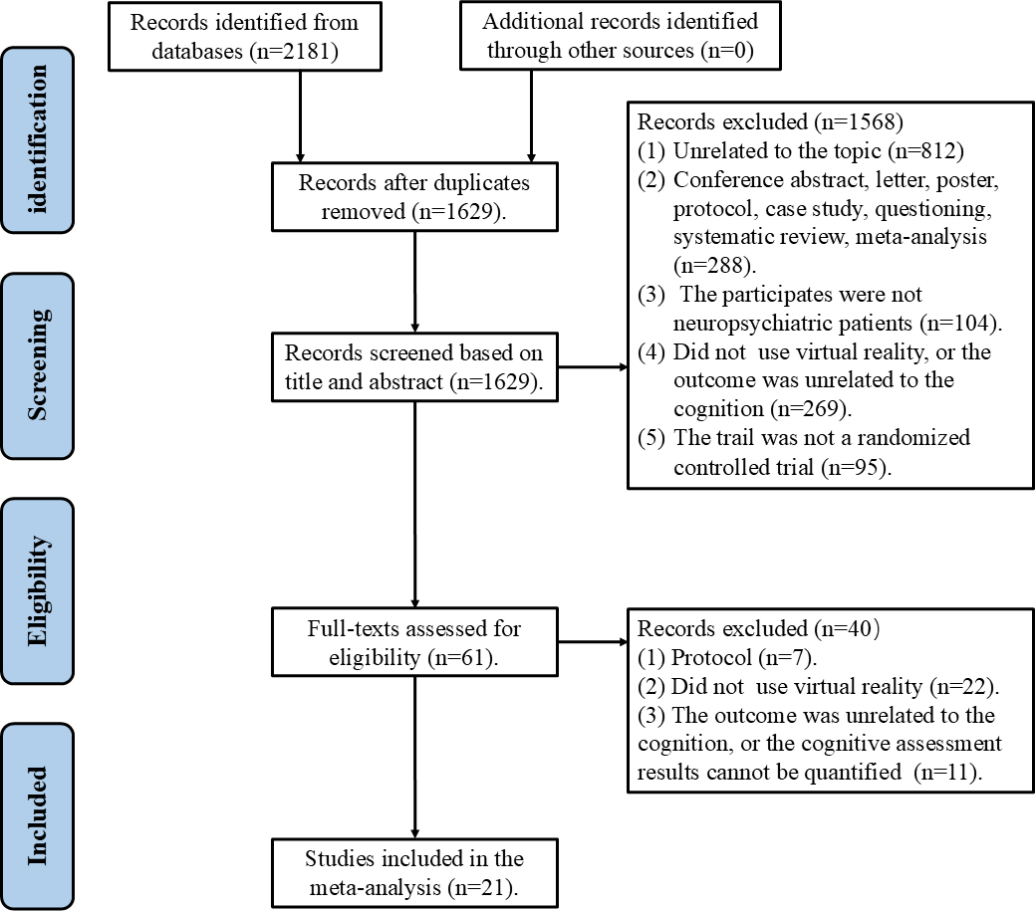
PRISMA flowchart. PRISMA: Preferred Reporting Items for Systematic Reviews and Meta-Analyses.

According to the 5 electronic databases, the initial search yielded a total of 2181 documents. Following a deduplication process, 552 redundant documents were eliminated. The exclusion of 1568 articles was based on a thorough evaluation of their titles and abstracts. Subsequently, the full texts of the remaining 61 articles underwent rigorous examination. Forty studies were excluded after full-text assessment for the following reasons: 7 were protocol studies, 22 did not employ VR technology, and 11 had outcomes unrelated to cognition or produced cognitive assessment results that could not be quantified. Consequently, 21 studies were included in the meta-analysis. A visual representation of the search and selection processes is provided in Figure 1. The unweighted Cohen κ values for study selection (0.86), data extraction (0.84), and risk of bias assessment (0.81) indicate an excellent level of interrater agreement, as per Cochrane Handbook criteria.

### Description of Studies in This Review

#### Study Characteristics

The salient attributes of the 21 studies that were incorporated into the analysis are documented in Multimedia Appendix 2. Each of these studies adhered to the rigorous design of RCTs and was published within the time frame spanning from 2010 to 2024. The review encompassed a total of 12 nations or regions, among which Korea, Hong Kong, China, and Taiwan, China, were notably included.

#### Characteristics of Patients

A total of 1051 patients were part of our research, with sample sizes varying from 17 [26] to 293 [17] individuals in each respective study. The mean age of the patients exhibited considerable variation, ranging from 14.81 (SD 1.74) years [27] to 77.35 (SD 6.75) years [18]. The ratio of males to females among the participants was nearly 1:2, with 356 males and 695 females; and the participants’ mean years of education spanned from 4.76 (SD 3.25) years [18] to 15.09 (SD 4.74) years [28] across the included studies. The participants’ disease types included schizophrenia (n=2, 9.5%), brain injury (n=2, 9.5%), Parkinson disease (n=3, 14.3%), mild cognitive impairment (n=8, 38.1%), stroke (n=3, 14.3%), and other neuropsychiatric disorders (n=3, 14.3%).

#### Characteristics of VR-Based Interventions

According to the goals and content of training or treatment, the intervention types in this review can be divided into 6 categories, involving cognitive rehabilitation training (n=8, 38.1%), immersive cognitive training (n=6, 28.6%), exergame-based training (n=4, 19.0%), music attention training (n=1, 4.8%), telerehabilitation and social functioning training (1, 4.8%), and vocational and problem-solving skills training (n=1, 4.8%).

For cognitive rehabilitation training, all exercises adopt a task-oriented learning model, meaning that specific goals and tasks are designed that patients need to complete to achieve rehabilitation objectives. Moreover, one of the characteristics of this type of intervention is the multimodal stimulation empowered by VR technology, implying that training is not limited to a single sensory input (such as visual) but integrates various sensory stimuli (such as visual, auditory, and tactile inputs) [26-33]. For instance, in the “ball and bird” activity of the IREX system (GESTURETEK HEALTH), users not only see the color of the ball but also experience different responses based on varying contact pressures [31]. Some training programs particularly emphasize combining physical movement with cognitive tasks, such as reciting poetry or doing mathematics problems while walking, promoting overall cognitive rehabilitation [32].

For immersive cognitive training, all exercises use highly immersive VR equipment [18,34-38]. The equipment primarily consists of head-mounted displays and wireless controllers or joysticks. Devices such as the Oculus Quest 2 (Meta Platforms, Inc) and HTC Vive (HTC Corporation) provide high-resolution visual output, capable of creating realistic virtual worlds. Wireless controllers or joysticks are used for interaction within the virtual environment, such as grabbing objects, operating machines, or navigating. Through haptic feedback (vibration or other physical signals), these devices simulate a genuine sense of touch, offering users a more direct operational experience within the virtual environment.

For exergame-based training, all exercises provide a variety of physical activities, including yoga and balance exercises, cycling control, and rowing training [17,39-41]. A key feature is the use of VR technology to achieve precise motion tracking. For instance, in rowing training, on-screen arrow indicators can help users understand how to correctly adjust their direction [41]; in cycling control games, wirelessly connected motion sensors can monitor the user’s riding posture, ensuring safety and effectiveness [17].

For music attention training, the study by Jeong et al [42] introduces an application based on a VR environment and the Unity platform (Unity Technologies Inc) that enhances attention and cognitive health through interactive music experiences. Participants drum according to the indication of falling colored balls on the screen, thereby exercising their focus, hand-eye coordination, and sense of rhythm while enjoying music. Regarding telerehabilitation and social functioning training, the research by Maggio et al [43] offers participants a platform enabled by VR technology that allows them to complete social challenges remotely via a smartphone. The core content of this training lies in simulating real-life social scenarios, such as initiating conversations and managing personal finances, with the aim of promoting cognitive rehabilitation and improving social skills. For vocational and problem-solving skills training, the study by Man et al [44] provides an artificial intelligence VR-based vocational training system training, which is based on VR technology and simulates various vocational skill exercises in an office environment setting, aiming to enhance cognitive functions. The aforementioned 6 types of interventions primarily simulate real-world scenarios, which can assist patients in transferring the skills they have learned to real-life environments, enhancing the transfer effect of cognitive improvements on activities of daily living. Meanwhile, all intervention types emphasize the importance of immediate interaction and feedback.

The technological implementations across 21 studies encompassed diverse hardware configurations. These included multiple VR headset models (HTC Vive, Oculus Rift CV1 [Meta Platforms, Inc], Oculus Quest 2, and Samsung Gear VR), video wall systems, portable computing devices, and supplementary rehabilitation equipment such as physical therapy instruments, Nintendo Wii balance boards (Nintendo Co, Ltd), and robotic exoskeletons. Complementing these hardware configurations, software solutions consisted of 3 main types: purpose-built applications developed for specific training protocols, modified commercial software packages, and unaltered off-the-shelf programs. Regarding methodological approaches, 6 studies adopted immersive methods and 15 studies used nonimmersive methods or unclear and ambiguous classifications. Notably, intervention sessions exhibited significant variation in frequency (2‐5 times weekly), session duration (15‐60 min), total session count (6‐40 sessions), and overall intervention duration (2 wk to 8 mo).

#### Characteristics of Controls

The control groups were subjected to random assignments, wherein they were allocated to either the conventional intervention or waitlist conditions for the entire study duration.

#### Outcome Measures

Multiple outcome measures were administered, and participants were assessed at various time points, with evaluations conducted after the intervention in all cases. Nine studies used the Montreal Cognitive Assessment scale to evaluate participants’ cognitive function, while 6 studies used the Mini-Mental State Examination scale for cognitive assessment. The remaining studies used various tools including the Loewenstein Occupational Therapy Cognitive Assessment-Geriatric, Repeatable Battery for the Assessment of Neuropsychological Status, Cognistat, the Wechsler Adult Intelligence Scale-Revised Block Design Test, the Das-Naglieri Cognitive Assessment System, and the Wechsler Memory Scale-Fourth Edition.

Both the Montreal Cognitive Assessment and Mini-Mental State Examination are widely used cognitive screening instruments that have demonstrated good reliability and validity across multiple studies [17,18,26,29,32-34,36,38-44]. The Loewenstein Occupational Therapy Cognitive Assessment-Geriatric is a cognitive assessment tool specifically designed for elderly individuals, focusing on cognitive functions relevant to daily activities [30]. Repeatable Battery for the Assessment of Neuropsychological Status is a standardized test battery intended for rapid screening and assessment of neuropsychological functioning in adults, encompassing 5 domains of cognition [28]. Cognistat is a clinically used tool for the quick screening and evaluation of an individual’s cognitive function, with a distinctive feature being its inclusion of an affective state screen and a preliminary assessment of the individual’s level of consciousness [31]. The Wechsler Adult Intelligence Scale-Revised Block Design Test aims to assess spatial visual ability, visual constructional skills, and problem-solving abilities [35]. Das-Naglieri Cognitive Assessment System is grounded in the PASS (Planning, Attention, Simultaneous, and Successive Processing) theory and is suitable for assessing cognitive functions in children and adolescents [27]. The Wechsler Memory Scale-Fourth Edition primarily evaluates memory functions within cognitive aspects [37].

### Risk of Bias

**Figure 2.**
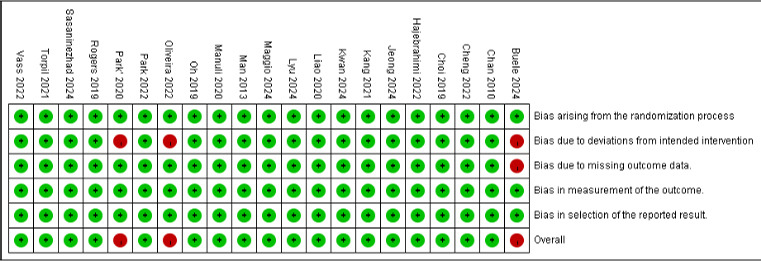
The risk of bias assessment from Cochrane RoB2 [17,18,26-44]. RoB2: Risk of Bias 2.

The methodological quality of the 21 selected studies is depicted in Figure 2. Most studies (n=18, 85.7%) were overall rated as low risk. However, the study by Buele et al [18] was assessed as high risk overall due to a lack of specific information on whether all participants adhered strictly to the intended intervention. Additionally, during the study, 5 participants withdrew. The text does not mention how these missing data were handled, leading to a high-risk rating due to deviations from intended intervention and bias arising from missing outcome data. Furthermore, the studies by Oliveira et al [26] and Park et al [38] were also rated as high risk overall because they did not specify if blinding was used and did not clearly state whether all participants adhered strictly to the intended intervention, resulting in a high risk of bias due to deviations from the intended intervention.

### Meta-Analysis Results

#### Overall Effect

**Figure 3.**
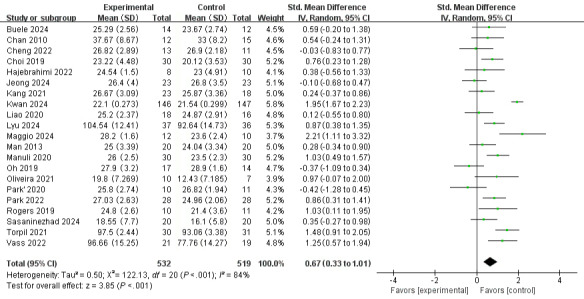
The overall effect of VR-based interventions on cognitive function in patients with neuropsychiatric disorders [17,18,26-44]. Std: standard; VR: virtual reality.

**Figure 4.**
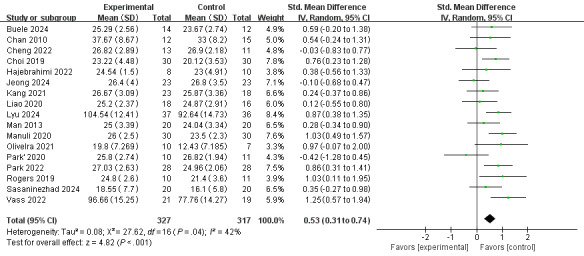
After removing 4 studies that may be sources of heterogeneity, the overall effect of VR-based interventions on cognitive function in patients with neuropsychiatric disorders [18,26-29,31,32,34-42,44] . Std: standard; VR: virtual reality.

Regarding cognitive function, a random-effects model was used, revealing a notable level of heterogeneity among the 21 included studies (*I*^2^=84%, *P*<.001). The meta-analysis’ overall effect indicated that interventions based on VR technology could improve cognitive functions in patients with neuropsychiatric disorders (standardized mean difference [SMD] 0.67, 95% CI 0.33-1.01, *z*=3.85, *P*<.001; Figure 3). For problems with heterogeneity >50%, we conducted a sensitivity analysis by removing the 4 studies [17,30,33,43] and recalculated the combined estimate on remaining studies, and a significant decrease in heterogeneity was found (*I*^2^ decreased from 84% to 42%). The overall effect of the meta-analysis demonstrated a similar result as above (SMD 0.53, 95% CI 0.31-0.74, *z*=4.82; *P*<.001), confirming the robustness of the conclusion. (Figure 4). After re-examining these 4 studies, we identified that the potential sources of heterogeneity may include disparities in baseline participant characteristics, divergent sample sizes, variability in assessment approaches, differences in intervention durations, etc.

#### Subgroup Analysis Based on Intervention Types

**Figure 5.**
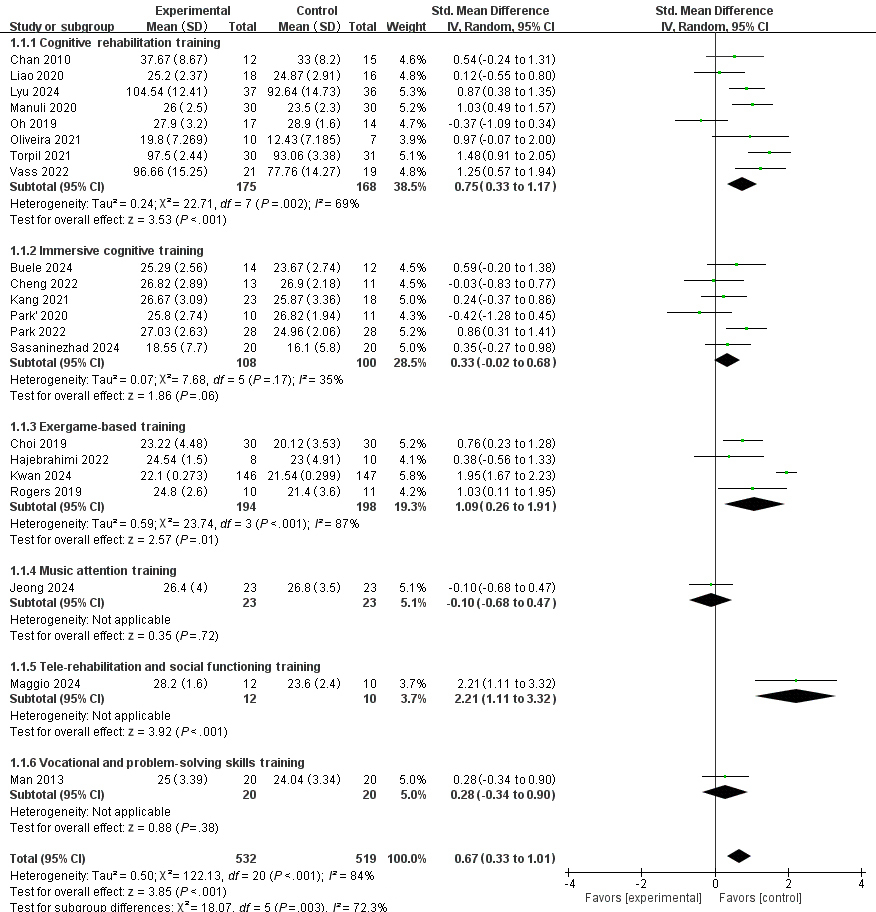
The effect of subgroup analysis based on intervention types [17,18,26-44]. Std: standard.

According to the types of interventions, the data were stratified into 6 distinct groups for subgroup analysis, including cognitive rehabilitation training, immersive cognitive training, exergame-based training, music attention training, telerehabilitation and social functioning training, and vocational and problem-solving skills training. The results showed that based on VR, cognitive rehabilitation training, exergame-based training, and telerehabilitation and social functioning training could significantly improve cognitive functions in patients with neuropsychiatric disorders (SMD 0.75, 95% CI 0.33-1.17, *z*=3.53, *P*<.001; SMD 1.09, 95% CI 0.26-1.91, *z*=2.57, *P*=.01; SMD 2.21, 95% CI 1.11-3.32, *z*=3.92, *P*<.001; respectively), whereas immersive cognitive training, music attention training, and vocational and problem-solving skills training do not yield significant improvements in cognitive functions for these patients (SMD 0.33, 95% CI –0.02 to 0.68, *z*=1.86, *P*=.06; SMD −0.1, 95% CI −0.68 to 0.47, *z*=0.35, *P*=.72; SMD 0.28, 95% CI −0.34 to 0.90, *z*=0.88, *P*=.38; respectively). Additionally, the groups of cognitive rehabilitation training and exergame-based training exhibited high heterogeneity, with *I*² values of 69% and 87%, respectively (Figure 5).

#### Subgroup Analysis Based on Disease Types

**Figure 6.**
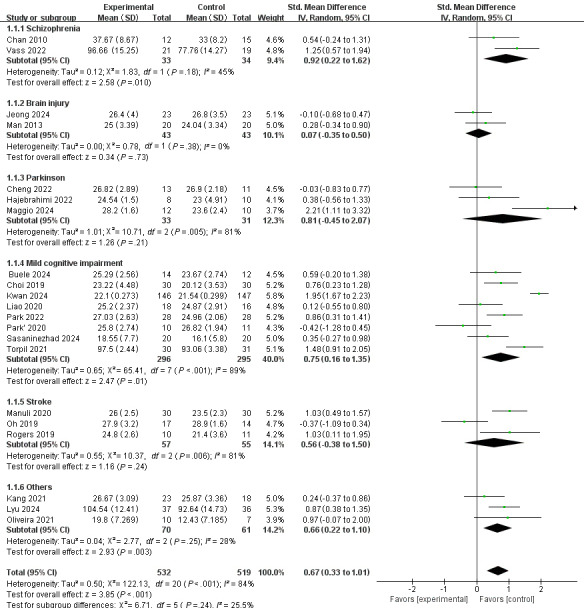
The effect of subgroup analysis based on disease types [17,18,26-44]. Std: standard.

To investigate the potential reasons for differences in the impact of various intervention types based on VR technology on the cognitive functions of patients with neuropsychiatric disorders, besides the reasons related to the intervention types, we also proposed to analyze potential causes from the perspective of the types of neuropsychiatric disorders. The data was divided into 6 subgroups for subgroup analyses: schizophrenia, brain injury, Parkinson disease, mild cognitive impairment, stroke, and others. We found that VR-based interventions significantly improved cognitive functions in individuals with schizophrenia, mild cognitive impairment, and other neuropsychiatric disorders (SMD 0.92, 95% CI 0.22-1.62, *z*=2.58, *P*=.01; SMD 0.75, 95% CI 0.16-1.35, *z*=2.47, *P*=.01; SMD 0.66, 95% CI 0.22-1.10, *z*=2.93, *P*=.003; respectively), but did not yield significant improvements for those with brain injuries, Parkinson disease, or stroke (SMD 0.07, 95% CI −0.35 to 0.50, *z*=0.34, *P*=.73; SMD 0.81, 95% CI −0.45 to 2.07, *z*=1.26, *P*=.21; SMD 0.56, 95% CI −0.38 to 1.50, *z*=1.16, *P*=.24; respectively). High heterogeneity was observed in the Parkinson disease, mild cognitive impairment, and stroke groups, with *I*² values of 81%, 89%, and 81%, respectively (Figure 6).

We found that among the 6 studies classified as immersive cognitive training, 5 focused on patients with mild cognitive impairment or predementia. Subgroup analyses consistently indicated that interventions based on VR technology could effectively improve cognitive function in these patients. Notably, the study by Cheng et al [34] targeted patients with Parkinson disease, and subgroup analyses showed that VR-based interventions had no effect on improving cognitive function in these patients; therefore, we speculated that this study may be a potential reason for the lack of statistically significant differences in the outcomes of immersive cognitive training. Subsequently, we conducted a sensitivity analysis by excluding the study by Cheng et al [34], which resulted in immersive cognitive training, showing statistically significant improvements in cognitive function. This further confirmed our hypothesis. Music attention training and vocational and problem-solving skills training both focused on patients with brain injuries, and subgroup analyses showed that VR-based interventions did not improve cognitive function in patients with brain injuries.

### Publication Bias

**Figure 7.**
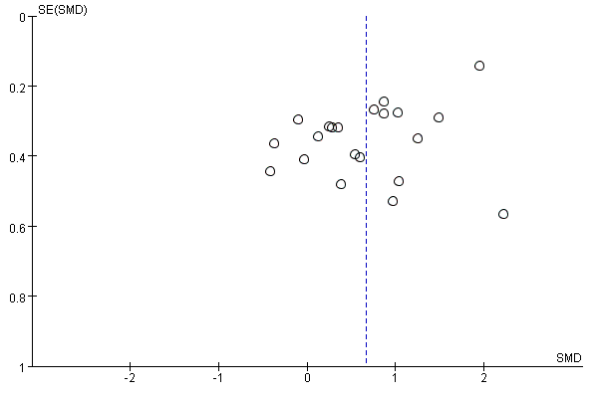
The funnel plot of publication bias. SMD: standardized mean difference.

To assess publication bias among the studies, a funnel plot is drawn. In this plot, the distribution of points is not entirely symmetrical, particularly on the left side, which could be an indication of publication bias. However, since most of the studies included in the meta-analysis report were smaller sample sizes, they may not accurately reflect publication bias (Figure 7).

## Discussion

### Principal Findings

The principal aim of this meta-analysis was to quantitatively assess the efficacy of VR-based interventions on cognitive function among individuals with neuropsychiatric disorders. Through subgroup analyses, we sought to elucidate the efficacy of distinct intervention types and to investigate potential reasons for differences in results. Our findings demonstrated that VR-based interventions yield a significant positive effect on cognitive function in patients with neuropsychiatric disorders. Specifically, subgroup analyses revealed that cognitive rehabilitation training, exergame-based training, and telerehabilitation and social functioning training were associated with improvements in cognitive performance. Conversely, immersive cognitive training, music attention training, and vocational and problem-solving skills training did not significantly enhance cognitive functions within this patient population. Further analysis from the standpoint of disease categorization suggested that the unique characteristics of conditions for Parkinson disease and brain injury may underlie the lack of statistical significance observed in the meta-analytic synthesis for immersive cognitive training, music attention training, and vocational and problem-solving skills training, which underscored the importance of considering the specific etiology and pathology of neuropsychiatric disorders when designing VR-based therapeutic interventions.

### Interpretation of Results

This study is the first to use quantitative analysis methods to evaluate the impact of VR-based interventions on cognitive functions in patients with neuropsychiatric disorders, providing preliminary evidence of potential efficacy in this therapeutic area. It represents an advancement in personalized medicine and the development of digital health tools. We included data from several relatively high-quality RCTs, revealing the efficacy and potential of VR-based interventions in improving cognitive functions, which are critical for the rehabilitation of neuropsychiatric disorders.

In our study, VR-based interventions featured immersive and highly interactive characteristics that can stimulate neuroplasticity in the brain. This property allows the brain to adapt to new information and skills by forming new synaptic connections or strengthening existing ones [45,46]. In VR environments, the simultaneous provision of visual, auditory, and tactile sensory inputs promotes synchronous activity among different sensory processing areas of the brain. This multisensory integration process is essential for constructing a coherent cognitive representation and can, to some extent, compensate for deficits caused by damage to certain sensory channels [47,48]. Different types of VR experiences can specifically activate particular regions of the brain. For instance, visual-spatial navigation tasks may increase activity in the hippocampus, whereas complex social interaction scenarios could impact the prefrontal cortex and other brain areas associated with higher cognitive functions [49,50]. This targeted stimulation may aid in repairing or optimizing the functions of these brain regions. Furthermore, VR-based interventions can also influence the brain’s reward system. When users complete challenges or achieve goals within a VR environment, they typically receive immediate feedback and reward signals, such as increased scores or virtual prizes. This type of positive reinforcement mechanism can activate the reward centers in the brain, such as the nucleus accumbens, triggering the release of dopamine, which in turn can increase motivation levels and enhance learning outcomes [51,52]. Another crucial point is that the VR-based interventions in this review primarily simulate real-world life scenarios, which is beneficial for improving the transfer effect of cognitive enhancements to activities of daily living. This approach aids in social reintegration and boosts the ability to live independently [53].

Through subgroup analysis of intervention types, we found that immersive cognitive training, music attention training, and vocational and problem-solving skills training based on VR technology did not improve cognitive functions in patients with neuropsychiatric disorders. This is inconsistent with previous research findings [54]. In previous studies, the aforementioned interventions primarily targeted patients with mild cognitive impairment or dementia; in contrast, our meta-analysis also included patients with Parkinson disease and brain injury. Second, subgroup analyses in this study revealed that exergame-based training was associated with cognitive improvement. Emerging evidence suggests that body position moderates the effectiveness of exergame-based training on global cognition, with stepping movement training in a predominantly standing position showing greater benefits compared to weight shifting or exergaming in a sitting position [55]. Consequently, the positive effects of exergame-based training on cognition may have been underestimated in our study. Notably, among the 4 exergame-based training studies included in this analysis, 3 used a seated position, while only 1 involved a standing position [17,39-41]. The limited sample size prevented further detailed and effective analysis. Therefore, future research should investigate the influence of body posture on the efficacy of different types of VR-based training to develop more comprehensive and effective cognitive intervention programs. Subgroup analysis by disease types revealed that patients with Parkinson disease and brain injury did not show significant cognitive improvements from VR-based interventions. After excluding the study on Parkinson disease [34] through sensitivity analysis, immersive cognitive training demonstrated statistically significant improvement effects. Parkinson disease, characterized by resting tremors, muscle rigidity, bradykinesia, and postural instability, can limit a patient’s ability to interact effectively within a VR environment, thereby reducing the efficacy of training [54]. For example, decreased fine motor control may impair the ability to complete tasks requiring hand-eye coordination [56]. Furthermore, abnormalities in cortico-basal ganglia circuits in Parkinson disease may exacerbate executive dysfunction, further hindering cognitive training outcomes [57]. Similarly, different types of brain injuries can lead to either localized or diffuse brain tissue damage. Localized injuries typically affect specific functional areas, whereas diffuse injuries disrupt broader neural networks. When critical neural pathways are severed, simple repetitive training may be insufficient to rebuild these connections, thereby limiting the effectiveness of VR-based cognitive training [58,59]. However, emerging evidence suggests that while VR-based training often presumes baseline executive functioning, such as planning and organization [57], recent trials demonstrate its adaptability to populations with neurological impairments. For example, Dubbeldam et al [60] reported that technology use did not lead to higher dropout rates, and adherence was significantly higher in tailored interventions. This finding aligns with Swinnen et al [61], who conducted a systematic review and found high attendance adherence rates even in institutionalized individuals with major neurocognitive disorder. Thus, the limited efficacy observed in our study may reflect limitations in the intervention design rather than being solely attributable to inherent neuroanatomical constraints. These findings underscore the importance of tailoring VR-based interventions to the specific needs and capabilities of patient populations, as well as optimizing the design to maximize therapeutic outcomes.

### Advantages and Prospects

The strength of this review lies in its novel use of meta-analysis to quantify the effects of VR-based interventions on cognitive functions in patients with neuropsychiatric disorders, significantly enhancing statistical power and providing a more comprehensive and robust estimate of intervention efficacy [62]. Simultaneously, subgroup analyses were conducted to evaluate the effects of different VR-based intervention types and to examine potential reasons for efficacy variations from the perspective of disease types. These findings help identify potentially effective VR interventions and technical components for improving cognitive functions in patients with neuropsychiatric disorders and suggest differences in responses to various interventions across patient groups. Consequently, this provides a scientific basis for the development of personalized treatment plans and promotes the precise application and advancement of VR technology in the rehabilitation of neuropsychiatric disorders.

### Limitations

This study acknowledges several limitations that merit consideration. First, the studies included in our review exhibited certain heterogeneity due to differences among participants’ baseline characteristics, sample sizes, assessment methods, intervention durations, etc. Second, only original research articles published in English were included, which may lead to language-related selection bias. Additionally, there may be some publication bias in this study. Last, despite using a broad search strategy across 5 major electronic databases, some studies have incomplete or entirely missing information sources. Therefore, our conclusions should be interpreted with caution.

### Implications for Research

As VR-based interventions advance within personalized medicine paradigms, future research must adopt structured, user-centered frameworks to align technological development with the specific needs, preferences, and capabilities of populations with neuropsychiatric disorders. Frameworks such as the Generative Co-Design Framework [63] and the Multidisciplinary Iterative Design of Exergames [64] emphasize participatory, iterative design involving end users, clinicians, and industry partners. These methodologies, exemplified by projects such as Brain-IT (information technology) help ensure that interventions are grounded in real-world usability and adherence while integrating established guidelines such as the Medical Research Council Framework for Complex Interventions [65,66]. This approach prioritizes multidisciplinary collaboration and continuous feedback to enhance translational impact. Beyond efficacy validation, research must investigate the mechanisms through which VR enhances cognitive function, such as neuroplasticity and attentional engagement, to identify potential commonalities or differential pathways across disorders. Mechanistic clarity will inform the optimization of VR components and protocols to better target therapeutic effects. Thus, by integrating user-centered design, mechanistic investigation, and technological innovation, VR has the potential to evolve into a robust, evidence-based tool for neuropsychiatric rehabilitation, optimizing recovery trajectories and long-term patient outcomes.

### Conclusions

This meta-analysis showed that interventions based on VR technology have the potential to improve cognitive functions in patients with neuropsychiatric disorders, particularly in areas such as cognitive rehabilitation training, exergame-based training, and telerehabilitation and social functioning training. The findings provide valuable evidence supporting the application of VR technology in the rehabilitation of neuropsychiatric disorders and highlight possible directions for future intervention strategies.

## Supplementary material

10.2196/67501 Multimedia Appendix 1 Sources searched and search strategies.

10.2196/67501 Multimedia Appendix 2 The characteristics of included studies.

10.2196/67501 Checklist 1 PRISMA 2020 checklist. PRISMA: Preferred Reporting Items for Systematic Reviews and Meta-Analyses.

## Abbreviations

AR: augmented reality
PASS: Planning, Attention, Simultaneous, and Successive Processing
PICOS: Patient or Population, Intervention, Comparison, Outcome, and Study
RCT: randomized controlled trial
RoB2: Risk of Bias 2
SMD: standardized mean difference
VR: virtual reality

## References

[1] Estes ML, McAllister AK (2016). Maternal immune activation: implications for neuropsychiatric disorders. Science.

[2] Fabrazzo M, Russo A, Camerlengo A (2021). Delirium and cognitive impairment as predisposing factors of COVID-19 infection in neuropsychiatric patients: a narrative review. Medicina (Kaunas).

[3] Huang YC, Lee Y, Lee CY (2020). Defining cognitive and functional profiles in schizophrenia and affective disorders. BMC Psychiatry.

[4] Liu G, Zhang X, Huo X, Li W (2022). Prevalence, influencing factors, and clinical characteristics of cognitive impairment in elderly patients with schizophrenia. Front Psychiatry.

[5] de Raykeer RP, Hoertel N, Blanco C (2019). Effects of depression and cognitive impairment on quality of life in older adults with schizophrenia spectrum disorder: results from a multicenter study. J Affect Disord.

[6] Foguet-Boreu Q, Sancho AG, Lopez JMS (2020). Association between cognitive impairment and cardiovascular burden in patients with severe mental disorder. Cogn Neuropsychiatry.

[7] Lepage M, Guimond S, Raedler T (2025). Strategies for achieving better cognitive health in individuals with schizophrenia spectrum: a focus on the canadian landscape: stratégies pour atteindre une meilleure santé cognitive chez les personnes souffrant du spectre de la schizophrénie: un regard sur le paysage canadien. Can J Psychiatry.

[8] Rodríguez-Blanco L, Lubrini G, Vidal-Mariño C, Ríos-Lago M (2017). Efficacy of cognitive rehabilitation of attention, executive functions, and working memory in psychotic disorders: a systematic review. Actas Esp Psiquiatr.

[9] Jensen JH, Knorr U, Vinberg M, Kessing LV, Miskowiak KW (2016). Discrete neurocognitive subgroups in fully or partially remitted bipolar disorder: associations with functional abilities. J Affect Disord.

[10] Lewandowski KE, Sperry SH, Cohen BM (2017). Treatment to Enhance Cognition in Bipolar Disorder (TREC-BD): efficacy of a randomized controlled trial of cognitive remediation versus active control. J Clin Psychiatry.

[11] Hu Y, Yuan X, Ye P (2023). Virtual reality in clinical nursing practice over the past 10 years: umbrella review of meta-analyses. JMIR Serious Games.

[12] Tieri G, Morone G, Paolucci S, Iosa M (2018). Virtual reality in cognitive and motor rehabilitation: facts, fiction and fallacies. Expert Rev Med Devices.

[13] Bohil CJ, Alicea B, Biocca FA (2011). Virtual reality in neuroscience research and therapy. Nat Rev Neurosci.

[14] Makransky G, Andreasen NK, Baceviciute S, Mayer RE (2021). Immersive virtual reality increases liking but not learning with a science simulation and generative learning strategies promote learning in immersive virtual reality. J Educ Psychol.

[15] Muurling M, de Boer C, Vairavan S (2023). Augmented reality versus standard tests to assess cognition and function in early Alzheimer’s disease. NPJ Digit Med.

[16] Kim SH, Cho SH (2022). Benefits of virtual reality program and motor imagery training on balance and fall efficacy in isolated older adults: a randomized controlled trial. Medicina (Kaunas).

[17] Kwan RYC, Liu J, Sin OSK (2024). Effects of virtual reality motor-cognitive training for older people with cognitive frailty: multicentered randomized controlled trial. J Med Internet Res.

[18] Buele J, Avilés-Castillo F, Del-Valle-Soto C, Varela-Aldás J, Palacios-Navarro G (2024). Effects of a dual intervention (motor and virtual reality-based cognitive) on cognition in patients with mild cognitive impairment: a single-blind, randomized controlled trial. J Neuroeng Rehabil.

[19] Kantola M, Ilves O, Honkanen S (2024). The effects of virtual reality training on cognition in older adults: a systematic review, meta-analysis, and meta-regression of randomized controlled trials. J Aging Phys Act.

[20] Riva G, Mancuso V, Cavedoni S, Stramba-Badiale C (2020). Virtual reality in neurorehabilitation: a review of its effects on multiple cognitive domains. Expert Rev Med Devices.

[21] Chen JW, Du WQ, Zhu K (2024). Network meta-analysis of the effects of different cognitive trainings on the cognitive function of patients with mild cognitive impairment. J Psychiatr Res.

[22] Page MJ, McKenzie JE, Bossuyt PM (2021). The PRISMA 2020 statement: an updated guideline for reporting systematic reviews. BMJ.

[23] Moon HJ, Han S (2022). Perspective: present and future of virtual reality for neurological disorders. Brain Sci.

[24] Sterne JAC, Savović J, Page MJ (2019). RoB 2: a revised tool for assessing risk of bias in randomised trials. BMJ.

[25] Veroniki AA, Jackson D, Viechtbauer W (2016). Methods to estimate the between-study variance and its uncertainty in meta-analysis. Res Synth Methods.

[26] Oliveira J, Gamito P, Souto T (2021). Virtual reality-based cognitive stimulation on people with mild to moderate dementia due to Alzheimer’s disease: a pilot randomized controlled trial. Int J Environ Res Public Health.

[27] Lyu S, Zhong S, Luo Y (2024). Effects of virtual reality-based cognitive training for adolescents with depressive episodes: a pilot randomized controlled study. Psychiatry Res.

[28] Vass E, Simon V, Csukly G, Fekete Z, Kis B, Simon L (2022). Virtual reality-based theory of mind intervention in schizophrenia: preliminary efficacy results. Compr Psychiatry.

[29] Manuli A, Maggio MG, Latella D (2020). Can robotic gait rehabilitation plus vrtual reality affect cognitive and behavioural outcomes in patients with chronic stroke? A randomized controlled trial involving three different protocols. J Stroke Cerebrovasc Dis.

[30] Torpil B, Şahin S, Pekçetin S, Uyanık M (2021). The effectiveness of a virtual reality-based intervention on cognitive functions in older adults with mild cognitive impairment: a single-blind, randomized controlled trial. Games Health J.

[31] Chan CLF, Ngai EKY, Leung PKH, Wong S (2010). Effect of the adapted virtual reality cognitive training program among Chinese older adults with chronic schizophrenia: a pilot study. Int J Geriatr Psychiatry.

[32] Liao YY, Tseng HY, Lin YJ, Wang CJ, Hsu WC (2020). Using virtual reality-based training to improve cognitive function, instrumental activities of daily living and neural efficiency in older adults with mild cognitive impairment. Eur J Phys Rehabil Med.

[33] Oh YB, Kim GW, Han KS (2019). Efficacy of virtual reality combined with real instrument training for patients with stroke: a randomized controlled trial. Arch Phys Med Rehabil.

[34] Cheng TC, Huang SF, Wu SY, Lin FG, Lin WS, Tsai PY (2022). Integration of virtual reality into transcranial magnetic stimulation improves cognitive function in patients with Parkinson’s disease with cognitive impairment: a proof-of-concept study. J Parkinsons Dis.

[35] Park JH (2022). Effects of virtual reality-based spatial cognitive training on hippocampal function of older adults with mild cognitive impairment. Int Psychogeriatr.

[36] Kang JM, Kim N, Lee SY (2021). Effect of cognitive training in fully immersive virtual reality on visuospatial function and frontal-occipital functional connectivity in predementia: randomized controlled trial. J Med Internet Res.

[37] Sasaninezhad M, Moradi A, Farahimanesh S, Choobin MH, Almasi-Dooghaee M (2024). Enhancing cognitive flexibility and working memory in individuals with mild cognitive impairment: exploring the impact of virtual reality on daily life activities. Geriatr Nurs.

[38] Park JH, Liao Y, Kim DR (2020). Feasibility and tolerability of a culture-based virtual reality (VR) training program in patients with mild cognitive impairment: a randomized controlled pilot study. Int J Environ Res Public Health.

[39] Hajebrahimi F, Velioglu HA, Bayraktaroglu Z, Helvaci Yilmaz N, Hanoglu L (2022). Clinical evaluation and resting state fMRI analysis of virtual reality based training in Parkinson’s disease through a randomized controlled trial. Sci Rep.

[40] Rogers JM, Duckworth J, Middleton S, Steenbergen B, Wilson PH (2019). Elements virtual rehabilitation improves motor, cognitive, and functional outcomes in adult stroke: evidence from a randomized controlled pilot study. J Neuroeng Rehabil.

[41] Choi W, Lee S (2019). The effects of virtual kayak paddling exercise on postural balance, muscle performance, and cognitive function in older adults with mild cognitive impairment: a randomized controlled trial. J Aging Phys Act.

[42] Jeong E, Ham Y, Lee SJ, Shin JH (2024). Virtual reality-based music attention training for acquired brain injury: a randomized crossover study. Ann N Y Acad Sci.

[43] Maggio MG, Luca A, Cicero CE (2024). Effectiveness of telerehabilitation plus virtual reality (Tele-RV) in cognitive e social functioning: a randomized clinical study on Parkinson’s disease. Parkinsonism Relat Disord.

[44] Man DWK, Poon WS, Lam C (2013). The effectiveness of artificial intelligent 3-D virtual reality vocational problem-solving training in enhancing employment opportunities for people with traumatic brain injury. Brain Inj.

[45] Gangemi A, De Luca R, Fabio RA (2023). Effects of virtual reality cognitive training on neuroplasticity: a quasi-randomized clinical trial in patients with stroke. Biomedicines.

[46] Ismail FY, Fatemi A, Johnston MV (2017). Cerebral plasticity: windows of opportunity in the developing brain. Eur J Paediatr Neurol.

[47] Friese U, Daume J, Göschl F, König P, Wang P, Engel AK (2016). Oscillatory brain activity during multisensory attention reflects activation, disinhibition, and cognitive control. Sci Rep.

[48] Benetti S, Zonca J, Ferrari A, Rezk M, Rabini G, Collignon O (2021). Visual motion processing recruits regions selective for auditory motion in early deaf individuals. Neuroimage.

[49] Salgado-Pineda P, Landin-Romero R, Portillo F (2016). Examining hippocampal function in schizophrenia using a virtual reality spatial navigation task. Schizophr Res.

[50] Ainsworth M, Sallet J, Joly O (2021). Viewing ambiguous social interactions increases functional connectivity between frontal and temporal nodes of the social brain. J Neurosci.

[51] Murty VP, Adcock RA (2014). Enriched encoding: reward motivation organizes cortical networks for hippocampal detection of unexpected events. Cereb Cortex.

[52] Mohebi A, Pettibone JR, Hamid AA (2019). Dissociable dopamine dynamics for learning and motivation. Nature New Biol.

[53] Buele J, Varela-Aldás JL, Palacios-Navarro G (2023). Virtual reality applications based on instrumental activities of daily living (iADLs) for cognitive intervention in older adults: a systematic review. J Neuroeng Rehabil.

[54] Kalia LV, Lang AE (2015). Parkinson’s disease. Lancet.

[55] Manser P, Herold F, de Bruin ED (2024). Components of effective exergame-based training to improve cognitive functioning in middle-aged to older adults - a systematic review and meta-analysis. Ageing Res Rev.

[56] Dahdal P, Meyer A, Chaturvedi M (2016). Fine motor function skills in patients with Parkinson disease with and without mild cognitive impairment. Dement Geriatr Cogn Disord.

[57] Benazzouz A, Mamad O, Abedi P, Bouali-Benazzouz R, Chetrit J (2014). Involvement of dopamine loss in extrastriatal basal ganglia nuclei in the pathophysiology of Parkinson’s disease. Front Aging Neurosci.

[58] Wolf JA, Koch PF (2016). Disruption of network synchrony and cognitive dysfunction after traumatic brain injury. Front Syst Neurosci.

[59] Bai L, Yin B, Lei S (2023). Reorganized hubs of brain functional networks after acute mild traumatic brain injury. J Neurotrauma.

[60] Dubbeldam R, Stemplewski R, Pavlova J (2024). Technology-assisted physical activity interventions for older people in their home-based environment: a scoping review. JMIR Preprints.

[61] Swinnen N, Vandenbulcke M, Vancampfort D (2022). Exergames in people with major neurocognitive disorder: a systematic review. Disabil Rehabil Assist Technol.

[62] Noma H, Sugasawa S, Furukawa TA (2024). Robust inference methods for meta-analysis involving influential outlying studies. Stat Med.

[63] Bird M, McGillion M, Chambers EM (2021). A generative co-design framework for healthcare innovation: development and application of an end-user engagement framework. Res Involv Engagem.

[64] Li Y, Munoz J, Mehrabi S, Middleton L, Cao S (2020). Multidisciplinary iterative design of exergames (MIDE): a framework for supporting the design, development, and evaluation of exergames for health.

[65] Manser P, de Bruin ED (2021). Making the best out of IT: design and development of exergames for older adults with mild neurocognitive disorder - a methodological paper. Front Aging Neurosci.

[66] Skivington K, Matthews L, Simpson SA (2021). A new framework for developing and evaluating complex interventions: update of Medical Research Council guidance. BMJ.

