# Supplementary material for: Efficacy of Virtual Reality–Based Interventions on Cognitive Function in Patients With Neuropsychiatric Disorders: Systematic Review and Meta-Analysis of Randomized Controlled Trials
Source: JMIR Serious Games. 2025 May 8;13:e67501. doi: 10.2196/67501 (PMC12080969; doi:10.2196/67501)
Supplement: Multimedia Appendix 1 [file games-v13-e67501-s001.docx]

| **Source** | **Search strategy** |
| --- | --- |
| 1.PubMed  [Most recent search performed: December 20, 2024] | 1. ("virtual reality"[MeSH Terms] OR "immersive environment"[All Fields] OR "VR"[All Fields] OR "serious game"[All Fields] OR "digital therapy"[All Fields] OR "computerized cognitive training"[All Fields])  2. ("cognitive function"[MeSH Terms] OR "cognition"[All Fields] OR "memory"[All Fields] OR "attention"[All Fields] OR "executive function"[All Fields])  3. ("neuropsychiatric disorder"[All Fields] OR "mental health"[All Fields] OR "psychiatric"[All Fields] OR "schizophrenia"[MeSH Terms] OR "bipolar disorder"[MeSH Terms] OR "depression"[MeSH Terms] OR "anxiety"[MeSH Terms] OR "dementia"[MeSH Terms])  4. ("randomized controlled trial"[Publication Type] OR "random allocation"[MeSH Terms] OR "clinical trial"[Publication Type] OR "placebo"[All Fields] OR "double blind method"[MeSH Terms] OR "single blind method"[All Fields])  5. 1 AND 2 AND 3 AND 4  6. filter: Humans  7. filter: Publication date from 2010/01/01 to 2024/12/20 |
| 2. Web of Science  [Most recent search performed: December 20, 2024] | TS=("virtual reality" OR VR OR "immersive technology" OR "serious game*" OR "digital intervention*") AND TS=("cognitive function" OR cognition OR memory OR attention OR "executive function" OR neuroplasticity) AND TS=("neuropsychiatric patient*" OR "mental health" OR schizophrenia OR "bipolar disorder" OR depression OR anxiety OR dementia) AND TS=(random* OR trial OR placebo OR "double blind*" OR "single blind*" OR "controlled study") AND DOCTYPE:("ar" OR "rv") AND LANGUAGE:English AND PUBYEAR:2010-2024 |
| 3. Medline  [Most recent search performed: December 20, 2024] | 1 exp Neuropsychiatric Disorders/  2 exp Mental Disorders/  3 exp Schizophrenia/  4 exp Bipolar Disorder/  5 exp Depression/  6 exp Anxiety Disorders/  7 exp Dementia/  8 exp Cognitive Dysfunction/  9 (neuropsychiatric* adj2 patient*).tw.  10 (mental adj2 health).tw.  11 or/1-10  12 exp Virtual Reality Therapy/  13 exp Video Games/  14 "virtual reality".tw.  15 VR.tw.  16 "serious game*".tw.  17 "digital intervention*".tw.  18 "computerized cognitive training".tw.  19 or/12-18  20 exp Cognition/  21 "cognitive function".tw.  22 cognition.tw.  23 memory.tw.  24 attention.tw.  25 "executive function".tw.  26 neuroplasticity.tw.  27 or/20-26  28 19 and 11 and 27  29 randomized controlled trial.pt.  30 controlled clinical trial.pt.  31 random*.ab.  32 placebo.ab.  33 trial.ab.  34 groups.ab.  35 or/29-34  36 (animals not (humans and animals)).sh.  37 35 not 36  38 28 and 37  39 (2010:2024).py.  40 38 and 39 |
| 4. EMBASE  [Most recent search performed: December 20, 2024] | 1 exp Neuropsychiatric Disorders/  2 exp Mental Disorders/  3 exp Schizophrenia/  4 exp Bipolar Disorder/  5 exp Depression/  6 exp Anxiety Disorders/  7 exp Dementia/  8 exp Cognitive Dysfunction/  9 (neuropsychiatric* adj2 patient*).tw.  10 (mental adj2 health).tw.  11 or/1-10  12 exp Virtual Reality Therapy/  13 exp Video Games/  14 "virtual reality".tw.  15 VR.tw.  16 "serious game*".tw.  17 "digital intervention*".tw.  18 "computerized cognitive training".tw.  19 (immersive adj2 technology).tw.  20 or/12-19  21 exp Cognition/  22 "cognitive function".tw.  23 cognition.tw.  24 memory.tw.  25 attention.tw.  26 "executive function".tw.  27 neuroplasticity.tw.  28 or/21-27  29 11 and 20 and 28  30 exp Randomized Controlled Trial/  31 exp Controlled Clinical Trial/  32 randomly.mp.  33 placebo.mp.  34 trial.mp.  35 "single-blind*".mp.  36 "double-blind*".mp.  37 or/30-36  38 29 and 37  39 limit to humans  40 limit to lang = "english"  41 limit to yr="2010 -Current"  42 38 and 39 and 40 and 41 |
| 5.Cochrane Library  [Most recent search performed: December 20, 2024] | #1 MeSH descriptor [Neuropsychological Test] explode all trees  #2 MeSH descriptor [Mental Disorders] explode all trees  #3 MeSH descriptor [Schizophrenia] explode all trees  #4 MeSH descriptor [Bipolar Disorder] explode all trees  #5 MeSH descriptor [Depressive Disorder] explode all trees  #6 MeSH descriptor [Anxiety Disorders] explode all trees  #7 MeSH descriptor [Dementia] explode all trees  #8 MeSH descriptor [Cognition Disorders] explode all trees  #9 (neuropsychiatric* adj patient*).ab,ti  #10 (mental adj health).ab,ti  #11 or/#1-#10  #12 MeSH descriptor [Virtual Reality Therapy] explode all trees  #13 MeSH descriptor [Video Games] explode all trees  #14 ("virtual reality" or VR).ab,ti  #15 "serious game*".ab,ti  #16 "digital intervention*".ab,ti  #17 "computerized cognitive training".ab,ti  #18 (immersive adj technology).ab,ti  #19 or/#12-#18  #20 MeSH descriptor [Cognition] explode all trees  #21 "cognitive function".ab,ti  #22 cognition.ab,ti  #23 memory.ab,ti  #24 attention.ab,ti  #25 "executive function".ab,ti  #26 neuroplasticity.ab,ti  #27 or/#20-#26  #28 (#11 AND #19 AND #27)  #29 MeSH descriptor [Randomized Controlled Trial] explode all trees  #30 MeSH descriptor [Controlled Clinical Trial] explode all trees  #31 random*.ab,ti  #32 placebo.ab,ti  #33 trial.ab,ti  #34 "single-blind*".ab,ti  #35 "double-blind*".ab,ti  #36 or/#29-#35  #37 (#28 AND #36)  #38 limit #37 to humans  #39 limit #38 to lang = "english"  #40 limit #39 to yr="2010 -Current" |
