# Supplementary material for: Efficacy of Virtual Reality–Based Interventions on Cognitive Function in Patients With Neuropsychiatric Disorders: Systematic Review and Meta-Analysis of Randomized Controlled Trials
Source: JMIR Serious Games. 2025 May 8;13:e67501. doi: 10.2196/67501 (PMC12080969; doi:10.2196/67501)
Supplement: Multimedia Appendix 2 [file games-v13-e67501-s002.docx]

Appendix 2. The characteristics of included studies.

| First Author/  Published  Year | Country/Region | Disease type | Age(experimental/control) (Mean [SD]) (years) | Education(experimental/control)  (Mean [SD]) (years) | Male/Female | Sample Size (experimental/control) | Intervention type | Intervention content | Control | Duration | Assessment time | Outcomes of cognitive function |  |
| --- | --- | --- | --- | --- | --- | --- | --- | --- | --- | --- | --- | --- | --- |
| Buele [1],2024 | Ecuador | Mild cognitive  impairment | 75.41 (5.76)/77.35 (6.75) | 5.53 (3.18)/ 4.76 (3.25) | 11/23 | 17/17 | Immersive Cognitive Training | Using immersive Oculus Quest 2 VR goggles and a wireless controller, participants undergo cognitive training in a virtual kitchen, where the task is to remember and locate cooking ingredients placed at random positions. | Conventional intervention | 6-week intervention, 2 times/week, 20min/session. | Post- intervention | MoCA-S |  |
| Cheng [2], 2022 | Taiwan, China | Parkinson | 71.4 (8.5)/ 71.6 (5.1) | NR | 13/11 | 13/11 | Immersive Cognitive Training | Using the immersive HTC Vive head-mounted display, the VR cognitive training scenarios were designed based on daily activities such as shopping at a supermarket, using an ATM, and driving. | Conventional intervention | 40min/day | Post- intervention | MoCA |  |
| Park [3], 2022 | Thailand | Mild cognitive  impairment | 71.93 (3.11)/ 72.04 (2.42) | 8.42 (4.23)/ 8.78 (4.13) | 23/33 | 28/28 | Immersive Cognitive Training | Using an immersive virtual reality environment, participants navigated with a joystick to complete tasks involving collecting gems based on clues and returning to the starting point. | on a waitlist without VR | A total of 24 sessions, 45 minutes a session, 3 days a week for 8 weeks. | Post- intervention | WAIS-BDT |  |
| Kang [4],2021 | Korea | Predementia | 75.48 (4.67)/ 73.28 (6.96) | 7.70 (4.10)/ 8.56 (4.83) | 12/29 | 23/18 | Immersive Cognitive Training | Utilizing fully immersive VR cognitive training, which includes multiple game-form tasks such as spot-the-difference games, finding paths based on memorized maps, capturing animals in a specific order, etc. | Conventional intervention | 2 times/week for one month | Post- intervention | MMSE |  |
| Sasaninezhad[5],2024 | Iran | Mild cognitive impairment | 70.30 (6.59)/ 69.10 (5.05) | NR | 19/21 | 20/20 | Immersive Cognitive Training | An immersive virtual reality was used to create simulated environments of a shopping mall and home settings. The tasks included recalling a shopping list, navigating aisles, locating and retrieving items, and completing transactions at the cashier. | Conventional intervention | A total of 10 sessions, 3 times /week, each lasting 30 minutes. | Post- intervention | WMS-IV |  |
| Park’ [6],2020 | Korea | Mild cognitive impairment | 71.80 (6.61)/ 69.45 (7.45) | 7.20 (3.61)/ 8.00 (2.90) | 7/14 | 10/11 | Immersive Cognitive Training | Using the immersive HTC Vive head-mounted display for VR training, the specific tasks include improving attention by shooting designated targets, enhancing memory by firing fireworks in numerical order, and improving logical thinking and numerical skills through simulating daily shopping activities, etc. | Usual care | 2 times/week, 30 minutes/time, for three months. | Post- intervention | K-MMSE |  |
| Manuli [7],2020 | Italy | Stroke | 48.0 (12.1)/ 40.1 (10.7) | 13.2 (2.9)/ 12.1 (3.1) | 25/35 | 30/30 | Cognitive Rehabilitation Training | Training with the Lokomat-Pro robotic exoskeleton, which includes an interactive VR screen, involves tasks that require patients to perform activities within a virtual environment, such as collecting items or avoiding obstacles. | Conventional rehabilitation treatment | 5 times/week, 1h/ time, for eight weeks. | Post- intervention | MoCA |  |
| Torpil [8],2021 | Turkey | Mild cognitive impairment | 70.12 (2.57)/ 70.30 (2.73) | NR | 25/36 | 30/31 | Cognitive Rehabilitation Training | Utilizing VR technology through the Microsoft Kinect for PC program, a series of games are conducted, including Boxing Trainer, Jet Run, Superkick, Air Challenge, etc. | Conventional rehabilitation | 45-minute sessions twice a  week for 12 weeks. | Post- intervention | LOTCA-G |  |
| Vass [9],2022 | Hungary | Schizophrenia | 36.71 (11.73)/42.47 (8.74) | 14.09 (2.46)/15.09 (4.74) | 22/20 | 21/21 | Cognitive Rehabilitation Training | The training utilizes virtual environments where participants interact with virtual characters controlled by the therapist. These interactions include complex dialogic elements such as irony, metaphors, and puns. | Conventional intervention | 1h individual sessions per week, for 9-session. | Post- intervention | RBANS |  |
| Chan [10],2010 | Hong Kong, China | Schizophrenia | 66.41 (6.20)/ 65.87 (5.54) | NR | 18/9 | 12/15 | Cognitive Rehabilitation Training | The IREX system, supported by VR technology, includes two activities: "Ball and Bird" (where users can touch balls of different colors flying in from the periphery using any part of their body; depending on the intensity of contact, the balls may either "burst" or "transform" into pigeons that fly away); and "Shark Bait" (where users navigate through the sea by tilting left or right or squatting down while they must avoid sharks and eels). | Usual care | 15 minutes/time, for 10 sessions. | Post- intervention | Cognistat (total) |  |
| Oliveira [11],2021 | Portugal | Alzheimer’s Disease | NR | NR | 5/12 | 10/7 | Cognitive Rehabilitation Training | Using VR technology, instrumental activities of daily living are simulated through a computer program, with scenarios including a virtual apartment and a virtual city; tasks include morning hygiene, shoe closet test, wardrobe test, etc. | Usual care | A total of 10 sessions, 2 times/week, for 2 months. | Post- intervention | MMSE |  |
| Lyu [12],2024 | China | Depressive episodes | 15.11 (1.51)/ 14.81 (1.74) | 8.41 (1.28)/ 8.11 (2.14) | 24/49 | 37/36 | Cognitive Rehabilitation Training | The VRCT system, displayed using the Barco OverView OLSF-721 full HD 3D stereoscopic LED video wall, comprises two training modules: an attention module and a working memory module, each containing five training tasks. | on a waitlist without VR | A total of 20 sessions, 1 time/day, for 30min, 3 times/ week. | Post- intervention | DN: CAS |  |
| Liao [13],2020 | Taiwan, China | Mild cognitive impairment | 75.5(5.2)/73.1(6.8) | 9.3 (3.8)/ 9.9 (2.1) | 11/23 | 18/16 | Cognitive Rehabilitation Training | Using VR technology and the Kinect system, cognitive tasks were designed, such as reciting poems while walking and turning, enumerating animals or flower names while crossing obstacles of varying heights, and practicing math calculations while strengthening lower extremity muscles. | Conventional intervention | A total of 36 sessions, 60min/time, for 12 weeks. | Post- intervention | MoCA |  |
| Oh [14], 2019 | Korea | Stroke | 57.4 (12.2)/ 52.6 (10.7) | 11.7 (2.8)/ 12.0 (3.5) | 21/10 | 17/14 | Cognitive Rehabilitation Training | Based on VR technology, the Joystima 3D manipulator simulates real-life tasks such as functional movements and interactive tasks. | Conventional intervention | 3 times/week, 30min/time, for 6 weeks. | Post- intervention | K-MMSE |  |
| Jeong [15],2024 | Korea | Brain injury | 50.4 (15.7)/ 43.0 (13.4) | 14.3 (2.1)/ 14.5 (2.4) | 18/5 | 12/11 | Music Attention Training | Using VR technology combined with computer running Unity software, participants can drum according to the indication of colored balls falling from the top of the screen, where changes in color, number, and time interval guide the target drum and rhythm pattern. | Conventional intervention | two 4-week phases, with 30-minute sessions, 5 times/week. | Post- intervention | MMSE |  |
| Maggio [16],2024 | Italy | Parkinson | 59.7 (9.7)/ 66.8 (6.5) | 13.1 (3.5)/ 11.7 (5.2) | 17/5 | 12/10 | Tele-rehabilitation and Social Functioning Training | Based on VR technology, using remote cognitive rehabilitation apps, participants complete social challenges (such as initiating conversations, managing finances, work and family) through their smartphones and receiving audio and video feedback. | Conventional intervention | 15min/day, 3 times/week, for 6 weeks. | Post- intervention | MoCA |  |
| Hajebrahimi[17],2022 | Turkey | Parkinson | 66.36 (8.04)/ 65.53 (9.93) | NR | NR | 8/10 | Exergame-based Training | The interactive game format, which combines VR technology, includes yoga games, strengthening games, and balance games, and provides visual and auditory feedback. | Conventional intervention | A total of 12 sessions, 60min/time, for 4 weeks. | Post- intervention | MoCA |  |
| Rogers [18],2019 | Australia | Stroke | 64.3 (17.4)/ 64.6 (12.0) | 13.5 (2.1)/ 12.5 (1.9) | 12/9 | 10/11 | Exergame-based Training | Based on VR technology, the Elements system uses customized surface computing and tangible interfaces. The game tasks include goal-directed games (such as Base Task, Random Base Task, Chase Task, Go/No-Go Task) and exploratory games (such as Mixer, Squiggles, Swarm), while providing enhanced audiovisual feedback. | Conventional intervention | One on one training for 30-40min/time, 3 times/week, for 4 weeks. | Post- intervention | MoCA |  |
| Kwan [19],2024 | Hong Kong, China | Mild cognitive impairment | 75.2 (7.1)/ 73.9 (6.6) | NR | 64/229 | 146/147 | Exergame-based Training | Using VR games, motion sensors are connected to the headset via a wireless protocol, allowing users to control virtual movement from a bike. The game features 8 themes that simulate daily activities for the elderly, such as finding bus stops, reporting lost items, shopping, and more. | on a waitlist without VR | 2 times/week, 1 hour/time, for 8 weeks. | Post- intervention | MoCA |  |
| Choi [20],2019 | Korea | Mild cognitive impairment | 77.27 (4.37)/ 75.37 (3.97) | NR | 9/51 | 30/30 | Exergame-based Training | For the VR-based kayak paddling training, arrows are displayed on the screen to indicate direction changes, helping participants understand and follow the movement direction of the virtual boat. A soft balanced foam is placed under the chairs to simulate the sensation of floating on water. | Conventional intervention | 2 times/week, 60 min/time, for 6 weeks. | Post- intervention | MoCA |  |
| Man [21],2013 | Hong Kong, China | Traumatic brain injury | NR | NR | NR | 20/20 | Vocational and Problem-Solving Skills Training | AIVTS training covers office item identification and placement, handling supplies like correction fluid and documents, using equipment such as printers, performing tasks like mail management, and addressing office safety and posture. It also supports interactive sessions with visual and auditory feedback. | Conventional intervention | 20-25min/time, 12 times. | Post- intervention | MMSE |  |

MoCA, the Montreal Cognitive Assessment; MoCA-S, Spanish version of the Montreal Cognitive Assessment; VR, virtual reality; WAIS-BDT, the Weschsler Adult Intelligence Scale-Revised Block Design Test; MMSE, the Mini-Mental State Examination; WMS-IV, the Wechsler Memory Scale- Fourth edition; K-MMSE, the Korean Mini-Mental State Examination; LOTCA-G, Loewenstein Occupational Therapy Cognitive Assessment-Geriatric; RBANS, Repeatable Battery for the Assessment of Neuropsychological Status; DN: CAS, the Das-Naglieri Cognitive Assessment System; SD, standard deviation; NR, not reported.

References:

[1] Buele J, Avilés-Castillo F, Del-Valle-Soto C, Varela-Aldás J, Palacios-Navarro G. Effects of a dual intervention (motor and virtual reality-based cognitive) on cognition in patients with mild cognitive impairment: a single-blind, randomized controlled trial. J Neuroeng Rehabil. Aug 1, 2024;21(1):130. [doi: 10.1186/s12984-024-01422-w] [Medline: 39090664]

[2] Cheng TC, Huang SF, Wu SY, Lin FG, Lin WS, Tsai PY. Integration of virtual reality into transcranial magnetic stimulation improves cognitive function in patients with Parkinson’s disease with cognitive impairment: a proof-of-concept study. J Parkinsons Dis. 2022;12(2):723-736. [doi: 10.3233/JPD-212978] [Medline: 34897103]

[3] Park JH. Effects of virtual reality-based spatial cognitive training on hippocampal function of older adults with mild cognitive impairment. Int Psychogeriatr. Feb 2022;34(2):157-163. [doi: 10.1017/S1041610220001131] [Medline: 32616109]

[4] Kang JM, Kim N, Lee SY, et al. Effect of cognitive training in fully immersive virtual reality on visuospatial function and frontal-occipital functional connectivity in predementia: randomized controlled trial. J Med Internet Res. May 6, 2021;23(5):e24526. [doi: 10.2196/24526] [Medline: 33955835]

[5] Sasaninezhad M, Moradi A, Farahimanesh S, Choobin MH, Almasi-Dooghaee M. Enhancing cognitive flexibility and working memory in individuals with mild cognitive impairment: exploring the impact of virtual reality on daily life activities. Geriatr Nurs. 2024;56:32-39. [doi: 10.1016/j.gerinurse.2023.12.008] [Medline: 38211369]

[6] Park JH, Liao Y, Kim DR, et al. Feasibility and tolerability of a culture-based virtual reality (VR) training program in patients with mild cognitive impairment: a randomized controlled pilot study. Int J Environ Res Public Health. Apr 27, 2020;17(9):3030. [doi: 10.3390/ijerph17093030] [Medline: 32349413]

[7] Manuli A, Maggio MG, Latella D, et al. Can robotic gait rehabilitation plus vrtual reality affect cognitive and behavioural outcomes in patients with chronic stroke? A randomized controlled trial involving three different protocols. J Stroke Cerebrovasc Dis. Aug 2020;29(8):104994. [doi: 10.1016/j.jstrokecerebrovasdis.2020.104994] [Medline: 32689601]

[8] Torpil B, Şahin S, Pekçetin S, Uyanık M. The effectiveness of a virtual reality-based intervention on cognitive functions in older adults with mild cognitive impairment: a single-blind, randomized controlled trial. Games Health J. Apr 2021;10(2):109-114. [doi: 10.1089/g4h.2020.0086] [Medline: 33058735]

[9] Vass E, Simon V, Csukly G, Fekete Z, Kis B, Simon L. Virtual reality-based theory of mind intervention in schizophrenia: preliminary efficacy results. Compr Psychiatry. Nov 2022;119:152350. [doi: 10.1016/j.comppsych.2022.152350] [Medline: 36272240]

[10] Chan CLF, Ngai EKY, Leung PKH, Wong S. Effect of the adapted virtual reality cognitive training program among Chinese older adults with chronic schizophrenia: a pilot study. Int J Geriatr Psychiatry. Jun 2010;25(6):643-649. [doi: 10.1002/gps.2403] [Medline: 19806599]

[11] Oliveira J, Gamito P, Souto T, et al. Virtual reality-based cognitive stimulation on people with mild to moderate dementia due to Alzheimer’s disease: a pilot randomized controlled trial. Int J Environ Res Public Health. May 16, 2021;18(10):5290. [doi: 10.3390/ijerph18105290] [Medline: 34065698]

[12] Lyu S, Zhong S, Luo Y, et al. Effects of virtual reality-based cognitive training for adolescents with depressive episodes: a pilot randomized controlled study. Psychiatry Res. Oct 2024;340:116144. [doi: 10.1016/j.psychres.2024.116144] [Medline: 39167866]

[13] Liao YY, Tseng HY, Lin YJ, Wang CJ, Hsu WC. Using virtual reality-based training to improve cognitive function, instrumental activities of daily living and neural efficiency in older adults with mild cognitive impairment. Eur J Phys Rehabil Med. Feb 2020;56(1):47-57. [doi: 10.23736/S1973-9087.19.05899-4] [Medline: 31615196]

[14] Oh YB, Kim GW, Han KS, et al. Efficacy of virtual reality combined with real instrument training for patients with stroke: a randomized controlled trial. Arch Phys Med Rehabil. Aug 2019;100(8):1400-1408. [doi: 10.1016/j.apmr.2019.03.013] [Medline: 31002812]

[15] Jeong E, Ham Y, Lee SJ, Shin JH. Virtual reality-based music attention training for acquired brain injury: a randomized crossover study. Ann N Y Acad Sci. Nov 2024;1541(1):151-162. [doi: 10.1111/nyas.15249] [Medline: 39476208]

[16] Maggio MG, Luca A, Cicero CE, et al. Effectiveness of telerehabilitation plus virtual reality (Tele-RV) in cognitive e social functioning: a randomized clinical study on Parkinson’s disease. Parkinsonism Relat Disord. Feb 2024;119:105970. [doi: 10.1016/j.parkreldis.2023.105970] [Medline: 38142630]

[17] Hajebrahimi F, Velioglu HA, Bayraktaroglu Z, Helvaci Yilmaz N, Hanoglu L. Clinical evaluation and resting state fMRI analysis of virtual reality based training in Parkinson’s disease through a randomized controlled trial. Sci Rep. May 16, 2022;12(1):8024. [doi: 10.1038/s41598-022-12061-3] [Medline: 35577874]

[18] Rogers JM, Duckworth J, Middleton S, Steenbergen B, Wilson PH. Elements virtual rehabilitation improves motor, cognitive, and functional outcomes in adult stroke: evidence from a randomized controlled pilot study. J Neuroeng Rehabil. May 15, 2019;16(1):56. [doi: 10.1186/s12984-019-0531-y] [Medline: 31092252]

[19] Kwan RYC, Liu J, Sin OSK, et al. Effects of virtual reality motor-cognitive training for older people with cognitive frailty: multicentered randomized controlled trial. J Med Internet Res. Sep 11, 2024;26:e57809. [doi: 10.2196/57809] [Medline: 39259959]

[20] Choi W, Lee S. The effects of virtual kayak paddling exercise on postural balance, muscle performance, and cognitive function in older adults with mild cognitive impairment: a randomized controlled trial. J Aging Phys Act. Apr 29, 2019;27(6):861-870. [doi: 10.1123/japa.2018-0020] [Medline: 31034303]

[21] Man DWK, Poon WS, Lam C. The effectiveness of artificial intelligent 3-D virtual reality vocational problem-solving training in enhancing employment opportunities for people with traumatic brain injury. Brain Inj. 2013;27(9):1016-1025. [doi: 10.3109/02699052.2013.794969] [Medline: 23662639]
